# Linking orthodontic treatment and sleep apnea among adult Indian patients

**DOI:** 10.6026/973206300200349

**Published:** 2024-04-30

**Authors:** Abhishek Kumar, Alok Kumar Gupta, Sikanderpal Kaur, Vinit Kumar Singh, Ashok Panika, Sandeep Singh

**Affiliations:** 1 Department of Orthodontics and Dentofacial Orthopedics, Dental college Azamgarh, Azamgarh, Uttar Pradesh, India; 2 Department of Orthodontics and Dentofacial Orthopedics, Vananchal Dental College, Farathiya Garwha, Jharkhand, India; 3 Newway Centre for Craniofacial Pain and Orthodontia, Chandigarh, India; 4 Department of Orthodontics and Dentofacial Orthopaedics, Government College of Dentistry, Indore, M.P., India; 5 Department of Orthodontics and Dentofacial Orthopaedics, Terna Dental College and Hospital, Navi Mumbai, Maharashtra, India

**Keywords:** Obstructive sleep apnea, orthodontic therapy

## Abstract

The differences in the effects of orthodontic treatment on airway and craniocervical posture in patients with OSA (obstructive sleep apnea) having skeletal class II high-angle malocclusion is of interest. Hence, 48 individuals with OSA and skeletal class II high-angle malocclusion were chosen from among all patients in need of orthodontic therapy. Every patients had CBCT (cone beam computed tomography) taken both before and after receiving orthodontic therapy. All parameters were assessed on the lateral cephalogram from CBCT in order to assess the indices of craniocervical posture, hyoid position, skeletal and dental conditions. Parameters of upper airway (position of hyoid) showed statistically significant increase in values after orthodontic treatments. Thus, there was increase in values of dimensions of upper airway, post orthodontic treatment. Hence, orthodontic therapy help improve the upper airway morphology and craniocervical posture in patients of OSA with hyperdivergent skeletal class II malocclusion.

## Background:

Obstructive sleep apnea (OSA) can worsen the patient's level of sleep at night and negatively impact a patient's day-to-day activities and work, and increase an adult's chance of developing major systemic disorders, such as diabetes, hypertension and coronary heart disease [1, 2-3]. Teens that have restricted airways are more prone to experience emotional turmoil, low performance in school, attention-deficit disorder and hyperactivity, restricted skeletal and physical growth, and cognitive impairment [4, 5, 6].The most common and the most difficult malocclusions in orthodontics is known as skeletal Class II high-angle malocclusion; it is characterized by vertical excessive growth of the mandible and sagittal inadequate growth [7, 8]. Patients typically experience limited airways along with unsatisfactory lateral appearances as a result of this malocclusion [9, 10]. Any treatment strategy that addresses down skeletal malformation should include forward mandibular rotation (FMR) in order to cope with the multitude of issues associated with hyperdivergent Class II sufferers [11, 12]. The growth potential of the skeletal effects of orthodontic therapy varies [13, 14]. The FMR mentioned above causes several growing patients' mandibles to grow more anteriorly, which improves their skeletal facial pattern [15, 16]. However, only a small number of adult patients have the indicated therapeutic benefits. Individuals with skeletal class II high angles are more likely to develop OSA [17, 18, 19, 20]. For this reason, orthodontic therapy and upper airway surveillance are essential for individuals with Class II high-angle malocclusion, especially those who are teens [21- 22, 23, 24]. A state known as craniocervical posture, which often represents the result of coordinating gravity and functional requirements, maintains the comparatively stable position of the cervical and craniofacial areas in both the inside and outside environments [13- 17]. There is a connection between both vertical and sagittal skeletal face morphology and craniocervical posture. In actuality, patients in skeletal Class II have a substantially bigger craniocervical angle compared to individuals with Class III due to a more lordotic arc of the backbone and an increased extension of the head [18- 24]. Furthermore, when comparing high-angle individuals to low-angle individuals, there is an apparent rise in the craniocervical angle in high angle patients [11-15]. Patient's attempt to achieve a wider airway is what causes the extension of the craniocervical posture, which helps to explain this occurrence [14-18]. As per the aforementioned notion, a number of researchers have documented notable alterations in craniocervical position subsequent to the alleviation of obstruction of the airways [19-21]. On the other hand, adjustments to the craniocervical position may have an impact on growth patterns. According to longitudinal studies, individuals with bigger craniocervical angles are more likely to have a vertical growth pattern, while those with lower craniocervical angles are more likely to have a horizontal growth pattern [21-25]. Nevertheless, there hasn't been much research done on how orthodontic therapy affects class II high-angle patients' alterations in craniocervical posture. Given that craniocervical posture and airway are impacted by improvements in craniofacial morphology, it was hypothesized that orthodontic therapy would result in a similar improvement in these areas in OSHS patients who have skeletal class II high-angle malocclusion [2-7]. Therefore, it is of interest to determine the differences in the effects of orthodontic treatment on airway and craniocervical posture in patients with OSA having skeletal class II high-angle malocclusion.

## Materials and Methods:

48 individuals with OSA and skeletal class II high-angle malocclusion were chosen from among all patients in need of orthodontic therapy between January 2021 and February 2024 for this retrospective analysis.

## Inclusion criteria:

[1] Patients who are at least eighteen years and above old

[2] High-angle pattern (MP-FH angle > 29°) with skeletal Class II malocclusion (ANB angle ≥ 4°)

[3] Patients whose treatment included the removal of 2 second premolars in mandible and 2 first premolars of maxilla

[4] Patients whose treatment included four bilaterally inserted micro-implants in the mandible and maxilla

[5] CBCT scans, both pre- and post-treatment, are accessible

## Exclusion conditions in order of presentation:

[1] A history of orthognathic surgery and/or orthodontic therapy

[2] Syndrome of temporo-mandibular joint diseases

[3] Past surgical upper airway experience

[4] Impairment of the function of the lip as well as palate (such as a cleft lip or palate)

Following the extraction of their second premolar of mandible and first premolar of maxilla, each patient received a pre-adjusted edgewise appliance with a 0.022-inch slot. The same orthodontist administered local anesthetic before implanting four mini-screws symmetrically through the buccal mucosa in the maxilla and mandible, between the first molars and second premolars. Four weeks following the micro-implant implantation, an elastic chain was used to produce a load of 150 g force. The patient's treatment took place over almost three years, with the goal of treating the Class I molar relation and Class I canine relationship.

Every patient had CBCT taken both before and after receiving orthodontic therapy. All CBCT data were exported in DICOM format and entered into Dolphin Imaging 11.95 software for 3D reconstruction and interpretation. By projecting the 3D reconstruction picture into the midsagittal plane from right to left, all parameters were assessed on the lateral cephalogram from CBCT in order to assess the indices of craniocervical posture, hyoidposition, skeletal and dental conditions. Using Dolphin Imaging software, the airway diameters were compared before and after therapy. The PP plane was aligned parallel to the horizontal plane in all photos to create a standard viewpoint. The upper airway border was defined by all planes corresponding to the PP plane. The laryngopharynx airway (LPA), glossopharynx airway (GPA) and velopharynx airway (VPA) are the three midsagittal portions of the upper airway that were physically separated. After the borders were established, Dolphin software was used to automatically determine the volume as well as minimum areas of LPA, GPA and VPA.

## Statistical analysis:

SPSS 26 program was used. The Kolmogorov-Smirnov test was used to evaluate whether the data had a normal distribution. Using the Wilcoxon signed rank test for non-normally generated variables and the paired t test for normally distributed data; a comparison of the pre-orthodontic treatment and post orthodontic treatment outcome variables was made. Pearson correlation analysis was used to measure items with a normal distribution, and Spearman rank correlation analysis was used to measure items with an irregular distribution. A difference that reached statistical significance was indicated by a p-value of less than 0.05, and the bilateral test level was set at α = 0.05.

## Results:

There was statistically significant improvement in most of the variables of craniofacial morphology after orthodontic therapy (Table 1). Parameters of upper airway (position of hyoid) showed statistically significant increase in values after orthodontic treatments (Table 2). There was statistically significant increase in values of dimensions of upper airway, post orthodontic treatment like LPA, GPA, VPA, Min GCSA and Min LCSA (Table 3). However, the values of VCSA didn't increase significantly (Table 3). Values of most of parameters of cervical inclination like increased significantly after orthodontic therapy (Table 4). Craniofacial inclination increased significantly after orthodontic treatment (Table 5). There was statistically significant increase in craniocervical inclination post therapy (Table 6).

## Discussion:

This retrospective study was aimed to determine the differences in the effects of orthodontic treatment on airway and craniocervical posture in patients with OSA having skeletal class II high-angle malocclusion. There was statistically significant increase in values of dimensions of upper airway, post orthodontic treatment like LPA, GPA, VPA, Min GCSA and Min LCSA in patients of OSA. However the values of VCSA didn't increased significantly. Position of hyoid bone showed statistically significant increase in values after orthodontic treatments. According to a study, the location of the hyoid bone and the size of the airway were improved in patients with Class II malocclusion when mandibular retrusion was adjusted using functional appliances [11- 18]. We saw a noteworthy increase in LPA, GPA, VPA, Min GCSA and Min LCSA in patients with OSA, which is consistent with the findings of the research mentioned above. Additionally, there was an anterior-inferior shift in the hyoid location, which could be related to the airway lengthening throughout development and growth [14- 21]. OSA is more common in those with bone class II high angles. Skeletal Class II high-angle malocclusions are the most prevalent and challenging malocclusions in orthodontics; they are typified by the mandible's excessive vertical growth and insufficient sagittal growth [13-16]. Because of this malocclusion, patients usually have restricted airways and undesirable lateral appearances [23]. Forward mandibular rotation (FMR) should be a part of any down skeletal deformity treatment plan in order to address the myriad of problems hyperdivergent Class II patients face [16- 19]. Thus, orthodontic therapy's impacts on the skeleton have differing growth potentials [20-24]. Several growing individuals see an improvement in their skeletal facial pattern as a result of the above-mentioned FMR, causing their mandibles to grow more anteriorly [18-21].

It is well known that the mandible's rotation in the opposite direction can cause tension in the suprahyoid muscles, which in turn causes the hyoid bone to position itself antero-superiorly, thereby expanding the upper airway's dimensions [2- 9]. Nonetheless, a research indicates that occlusal plane management does not yield a statistically significant improvement in the dimensions of the pharynx after treating adult patients with hyperdivergent skeletal Class II malocclusion [14-21]. Our study's results are in not in agreement with the findings presented with this research. The volume and minimum cross-sectional area of the adult group's upper airway considerably expand along with the occlusal plane and mandibular plane's reduced angles. Similarly the hyoid location was dramatically pushed forward. There was statistically significant increase in craniocervical inclination post therapy. Longitudinal investigations have demonstrated that a vertical growth pattern is more common in people with larger craniocervical angles, whereas a horizontal growth pattern is more common in those with smaller craniocervical angles [14- 17]. However, less study has been done on the effects of orthodontic therapy on changes in craniocervical posture in class II high-angle patients. It was hypothesized that orthodontic therapy would provide a similar improvement in craniocervical posture and airway in OSA patients with skeletal class II high-angle malocclusion, since these areas are affected by improvements in craniofacial morphology [11-15]. According to the literature [19-23], patients' mandibles will adapt forward during their development and growth, and they may migrate even further forward as the OP plane's inclination decreases [25]. This study hypothesizes that after orthodontic therapy, the upper airway will improve more noticeably due to this mandibular advancement.

The cervical and craniofacial regions are kept in a somewhat steady position in both indoor and outdoor contexts by a condition known as craniocervical posture, which frequently results from balancing gravity and functional requirements [9- 14]. There is a relationship between craniocervical position and the morphology of the vertical and sagittal skeleton of the face. As a result of a greater lordotic arc in the backbone and an enhanced extension of the head, patients in skeletal Class II actually have a significantly larger craniocervical angle than those in Class III [17- 23]. Moreover, there appears to be an increase in the craniocervical angle in high angle patients when comparing them to low angle persons [21]. To assist explain this phenomenon; a study suggests that the patient's attempt to achieve a broader airway is what leads to the expansion of the craniocervical position [18-24]. According to the previously indicated theory, some investigators have reported significant changes in craniocervical posture when airway blockage has been relieved [19-23]. However, changes to the craniocervical location could affect the way that an organism grows. It was postulated that patients with upper airway obstruction and high angle will extend their cervical column as a whole rather than only extending their head forward in order to achieve enough airflow [11-18]. This theory may have its roots in the fact that OSA patients with high angles cannot have their craniofacial and craniocervical postures extended to a large degree without compromising their horizontal visual axis [21-24]. Furthermore, this study's findings showed a correlation between the middle cervical column's inclination and the hyoid position parameter. In order to compensate, cervical extension may help move the hyoid bone away from the posterior pharyngeal wall, allowing the blocked airways to be released [25].

## Conclusion:

Data shows that orthodontic therapy help improve the upper airway morphology and craniocervical posture in patients of OSA with hyperdivergent skeletal class II malocclusion.

## Figures and Tables

**Table 1:** Comparison of parameters of craniofacial morphology before orthodontic treatment and after orthodontic treatment

**Variables**	**FMA(°)**	**SNA(°)**	**SNB(°)**	**ANB(°)**	**OP-FH(°)**	**Sum(°)**	**NBa-PtGn (mm)**	**Pog'-N'TVL(mm)**
Pre-treatment	36.32±4.66	81.51±3.20	75.46 ±2.57	7.15±1.33	14.4± 3.14	403.90±2.82	80.26±1.82	-8.03±2.01
Post-treatment	33.72±4.91	81.34±2.44	77.96±2.06	4.50±1.75	12.49±4.55	401.44±4.16	81.54±1.26	-4.65±3.26
P value	<0.001**	0.806	0.005*	<0.001**	0.027*	<0.001**	0.049*	0.014*

**Table 2:** Comparison of parameters of upper airway (position of hyoid bone) before orthodontic treatment and after orthodontic treatment

	**H-MP(mm)**	**H-FHP(mm)**	**H-C3VP(mm)**
Pre-treatment	11.21±3.71	80.66 ±5.20	29.15±2.58
Post-treatment	14.41±4.62	86.84 ±6.54	32.72±4.73
P value	0.033*	0.002**	0.037*

**Table 3:** Comparison of parameters of upper airway (dimensions of upper airway) before and after orthodontic treatment in adolescent patients

	**VPA(mm3)**	**GPA(mm3)**	**LPA(mm3)**	**MinVCSA(mm2)**	**MinGCSA(mm2)**	**MinLCSA(mm2)**
Pre-treatment	7345.00±5113.73	4169.79±216.49	2504.66±158.26	69.69±49.69	71.02±30.12	67.61±23.36
Post-treatment	10,933.18±2732.24	6603.05±2150.97	4937.41±2698.57	116.60±66.21	119.24±29.69	106.37±38.6
Pvalue	0.003**	0.007**	0.003**	0.061	0.002**	0.026*

**Table 4:** Comparison of variables of cervical inclination before and after orthodontic treatment in adolescent patients

	**CVT/EVT(°)**	**OPT/Ver(°)**	**CVT/Ver(°)**	**EVT/Ver(°)**
Pre-treatment	3.61±7.43	-7.61±5.27	-11.61±5.93	-13.94±6.99
Post-treatment	0.93±7.21	-4.79±4.66	-7.03±4.69	-9.94±7.46
Pvalue	0.549	0.029*	0.003**	0.022*

**Table 5:** Comparison of variables of craniofacial inclination before and after orthodontic treatment in adolescent patients

	**SN/Ver(°)**	**PP/Ver(°)**
Pre-treatment	97.61±5.07	87.19±4.59
Post-treatment	99.44±5.69	90.69±3.49
P value	0.019*	0.004**

**Table 6:** Comparison of variables of craniocervical inclination before and after orthodontic treatment in adolescent patients

	**SN-CVT(°)**	**SN-OPT(°)**
Pre-treatment	107.93±6.79	104.09±5.37
Post-treatment	106.26±6.26	103.11±4.10
P value	0.009**	0.425
